# The Impact of Cytokines in Coronary Atherosclerotic Plaque: Current Therapeutic Approaches

**DOI:** 10.3390/ijms232415937

**Published:** 2022-12-14

**Authors:** Panagiotis Tsioufis, Panagiotis Theofilis, Konstantinos Tsioufis, Dimitris Tousoulis

**Affiliations:** First Department of Cardiology, “Hippokration” General Hospital, University of Athens Medical School, 11527 Athens, Greece

**Keywords:** atherosclerosis, coronary artery disease, inflammation, interleukin, tumor necrosis factor

## Abstract

Coronary atherosclerosis is a chronic pathological process that involves inflammation together with endothelial dysfunction and lipoprotein dysregulation. Experimental studies during the past decades have established the role of inflammatory cytokines in coronary artery disease, namely interleukins (ILs), tumor necrosis factor (TNF)-α, interferon-γ, and chemokines. Moreover, their value as biomarkers in disease development and progression further enhance the validity of this interaction. Recently, cytokine-targeted treatment approaches have emerged as potential tools in the management of atherosclerotic disease. IL-1β, based on the results of the CANTOS trial, remains the most validated option in reducing the residual cardiovascular risk. Along the same line, colchicine was also proven efficacious in preventing major adverse cardiovascular events in large clinical trials of patients with acute and chronic coronary syndrome. Other commercially available agents targeting IL-6 (tocilizumab), TNF-α (etanercept, adalimumab, infliximab), or IL-1 receptor antagonist (anakinra) have mostly been assessed in the setting of other inflammatory diseases and further testing in atherosclerosis is required. In the future, potential targeting of the NLRP3 inflammasome, anti-inflammatory IL-10, or atherogenic chemokines could represent appealing options, provided that patient safety is proven to be of no concern.

## 1. Introduction

Coronary artery disease remains the leading cause of death in developed countries [1], despite the recent advances in its pharmacological and interventional management. The sudden rupture of the unstable atherosclerotic plaque and the subsequent platelet aggregation and thrombosis or thromboembolism is the pathologic hallmark of coronary artery disease and subsequent detrimental cardiovascular events [2]. Throughout the process of atherosclerosis, and especially in the formation and rupture of the vulnerable plaque, inflammatory cells and factors, such as cytokines, are involved [3]. As early as the mid-19^th^ century, Carl von Rokitansky and Rudolf Virchow detected cellular inflammatory changes in the atherosclerotic vessel walls, indicative of the role of inflammation in the disease development and progression. During recent years, research on atherosclerosis has been intense, highlighting the critical impact of inflammatory mediators in the establishment of a low-grade, sterile inflammatory state. Cytokines have been central in this regard, pointing to their potential as atherosclerotic disease biomarkers and even therapeutic targets, through cytokine-based treatment in atherosclerotic disease [4]. Therefore, in this review, we aimed to study the pathophysiology behind unstable atherosclerotic plaque, focusing on the role of inflammatory cytokines, and provide the latest data regarding the use of cytokines as therapeutic targets for the treatment of unstable atherosclerotic plaque.

## 2. Pathophysiology of the Atherosclerotic Plaque

Atherosclerotic plaque formation mainly depends on endothelial dysfunction, abnormal lipid metabolism, and inflammation. In the presence of genetic susceptibility, cardiovascular risk factors (hypertension, diabetes mellitus, smoking, inflammation), or low shear stress, there is augmented oxidative stress which promotes endothelial dysfunction, the initial step of atherogenesis [5]. The vascular endothelium represents an abundant organ lining blood vessels that is critical to atherosclerosis development and progression. In a healthy state, endothelial cells are responsible for regulating essential processes, such as the regulation of vascular tone, thrombosis and fibrinolysis, vascular inflammation and remodeling [6]. The main regulator of endothelial function is nitric oxide (NO), which possesses vasorelaxant, anti-thrombotic, anti-proliferative, and anti-inflammatory properties. However, when endothelial cells become activated, there is a change towards an atheroprone phenotype with the promotion of vasoconstriction, thrombosis, leukocyte mobilization-migration, and vascular smooth muscle cell (VSMC) proliferation [6]. This stems from an impaired bioavailability of NO due to an increased degradation paired with diminished production [6]. Uncoupling of endothelial NO synthase (eNOS), as a result of asymmetric dimethylarginine upregulation (endogenous eNOS inhibitor), oxidation of tetrahydrobiopterin (BH_4_), or failure of BH_4_ salvage pathway, represents the critical process contributing to impaired NO bioavailability and reactive oxygen species (ROS) production [6].

In the presence of a dysfunctional endothelial layer, the increased permeability aids the penetration of apolipoprotein B-containing lipoproteins up to approximately 70 nm in diameter, namely low-density lipoprotein (LDL), in the vessel wall [7]. LDL is a heterogeneous molecule that is responsible for the transport of insoluble cholesterol. Four LDL categories have been described, with small-dense LDL (sdLDL) being the most atherogenic. The influx of LDL particles is no longer considered a passive process. Their transcytosis depends on caveolae, scavenger receptor B1 (SR-B1)-dedicator of cytokinesis 4 (DOCK4) coupling, and activin receptor-like kinase 1 (ALK1) [7]. Subsequently, they are retained in the vessel wall owing to the interaction of the positively charged arginine and lysine of apoB100 with the negatively charged sulfate and carboxylic acid of the arterial wall proteoglycans. LDL can then undergo multiple modifications, including oxidation, electronegativity, desylation, glycation, and self-association [8]. Oxidized LDL (oxLDL) molecules bind to the lectin-like oxLDL receptor-1 (LOX-1) that is located on the surface of vascular smooth muscle cells and macrophages, leading to the formation of foam cells [9]. Moreover, LDL particles can form complexes with proteoglycans and glycosaminoglycans of the arterial wall, which may also be uptaken by macrophages. Furthermore, oxLDL could secrete bioactive lipids with local and systemic effects. Additionally, cholesterol crystal formation by LDL can promote NLR-family pyrin domain-containing protein 3 (NLRP3) inflammasome activation. Finally, LDL can induce innate and adaptive immune responses. On the other hand, the atheroprotective high-density lipoprotein (HDL) can remove cholesterol from foam cells by the stimulation of reverse cholesterol transport [10]. Additionally, HDL can enhance endothelial cell function, prevent LDL oxidation, and adhesion molecule expression. Last but certainly not least, inflammation orchestrates a detrimental cascade involving endothelial activation, monocyte adhesion and subendothelial transmigration, platelet activation, and coagulation that participates in atherogenesis [6,11,12,13,14]. Current concepts in the inflammatory concept of atherosclerosis include the atheroprone potential of NLRP3 inflammasome and neutrophil extracellular traps (NETs) [15,16].

The progression of atherosclerotic plaques into unstable mainly depends on the formation of so-called thin cap fibroatheromas (TCFAs) [17]. Firstly, as foam cells continue to accumulate due to the uptake of modified LDL by VSMCs and macrophages, a lipid-rich core begins to form. Secondly, the presence of defective efferocytosis of cellular debris from apoptotic VSMCs and macrophages leads to a lipid-rich necrotic core. Perhaps most importantly, the decreased collagen synthesis and the increased collagen degradation based on the action of interferon-γ (IFN-γ) and matrix metalloproteinases (MMPs), respectively, promotes the thinning of the fibrous cap that surrounds the lipid-rich necrotic core. Microscopic or spotty calcification is another factor that is associated with plaque vulnerability [18]. Ultimately, plaque rupture and exposure of thrombogenic plaque substances, such as tissue factor, to coagulation proteins of the blood induce thrombus formation, clinically translating to acute ischemic events. It should be noted that superficial plaque erosion is an increasingly common phenotype of plaque progression and disruption nowadays. Plaque erosion is based on disturbed endothelial shear stress, endothelial-to-mesenchymal transition, and ultimately endothelial cell apoptosis [19]. Moreover, the recruited leukocytes also undergo apoptosis and NETosis, promoting platelet activation and the formation of platelet-rich thrombi [19].

## 3. Cytokines in Coronary Atherosclerosis

**Figure 1:**
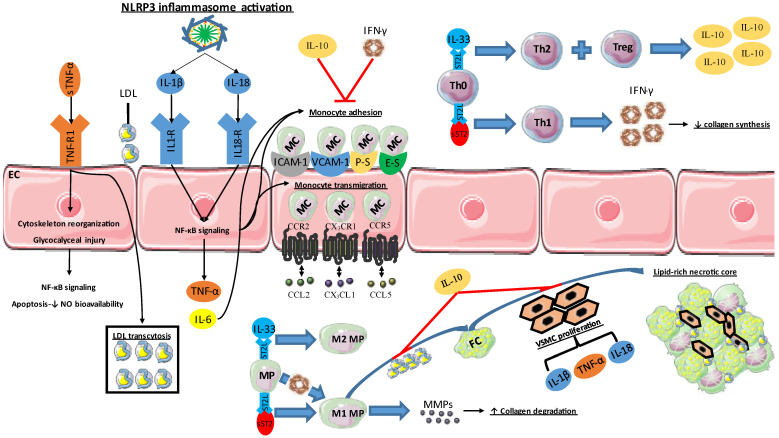
Role of major cytokines and chemokines in atherosclerosis initiation and progression. Cytokines are involved in critical steps of the atherosclerotic cascade, such as endothelial cell (EC) activation and dysfunction, low-density lipoprotein (LDL) transcytosis, monocyte adhesion and transmigration. Moreover, they are important mediators of macrophage (MP) phenotype and T helper 0 (Th0) differentiation. Additionally, they are involved in extracellular matrix remodeling, vascular smooth muscle cell (VSMC) proliferation. Ultimately, they contribute to the formation of a lipid-rich necrotic core surrounded by a thin fibrous cap. TNF: tumor necrosis factor; IL: interleukin; NF-κB: nuclear factor-kappaB; NO: nitric oxide; MC: monocyte; IFN: interferon; FC: foam cell; ST2L: suppression of tumorigenicity 2 ligand; MMP: matrix metalloproteinase; ICAM-1: intracellular adhesion molecule-1; VCAM-1: vascular cell adhesion molecule-1; CCL: chemokine (C-C motif) ligand; CCR: C-C chemokine receptor; CXCL: chemokine (C-X-C motif) ligand; CXCR: CXC chemokine receptors.

Cytokines are small (15–20-kD), hormone-like, soluble proteins that act as mediators aiding the communication between immune cells and neighboring or distant organs [20]. Cytokines perform a critical mission in the immune system by modulating the humoral and cell-based immune responses to infection and inflammation. They are produced by immune cells, namely macrophages, B lymphocytes, T lymphocytes, mast cells and by endothelial cells, platelets, fibroblasts, and some stromal cells [21]. Different cells may produce the same cytokine. The cytokines family includes interleukins (ILs), chemokines, interferons (IFNs), and tumor necrosis factors (TNFs). Below we discuss the role and the importance of the most extensively investigated cytokines in coronary atherosclerosis (Figure 1 and Table 1).

### 3.1. Interleukins

Interleukins appear to play the most important role in the atherosclerotic process and are synthesized and secreted by white blood cells, mainly CD4+ T helper cells, as well as monocytes, macrophages, and endothelial cells.

#### 3.1.1. IL-1 Family

The IL-1 family consists of 11 members encoded by 11 distinct genes, of which IL-1, IL-1 receptor antagonist (IL-1Ra), IL-18, and IL-33 are the most well-characterized. IL-1, in particular, has two subtypes (IL-1α, IL-1β) which bind to type 1 IL-1 receptor and take part in the regulation of immune responses, inflammatory reactions, and hematopoiesis [22]. Since IL-1α is mostly membrane-bound, it functions primarily locally rather than systemically. On the other hand, the main circulating version of IL-1 is IL-1β.

Critical to the production of IL-1β is the NLRP3 inflammasome. It consists of the innate immune sensor NLRP3, the adaptor molecule ASC, and the effector protease pro-caspase-1 [23]. The first step of NLRP3 inflammasome activation consists of priming by endogenous cytokines, thus upregulating NLRP3 and pro-IL1β through nuclear factor-kappaB (NF-κB) activation. In the setting of increased ion fluxes (K^+^, Cl^−^, Ca^2+^, Na^+^), lysosomal leakage, mitochondrial dysfunction, or oxidative stress, the second step of NLRP3 inflammasome activation ensues [24]. Noncanonical and alternative activation, through cytosolic lipopolysaccharide or Toll-like receptor-4 (TLR4)–TIR-domain-containing adaptor-inducing interferon-β (TRIF)–receptor-interacting serine/threonine-protein kinase 1 (RIPK1)–Fas-associated protein with death domain (FADD)–CASP8 signaling, has also been described. However, the effect of NLRP3 inflammasome on endothelial function depends mostly on the augmented reactive oxygen species (ROS) production. ROS further aggravate endoplasmic reticulum stress, which, in turn, promotes NLRP3 inflammasome activation through p38 mitogen-activated protein kinase (MAPK) pathway, Jun-N-terminal kinase (JNK) signaling, X-box-binding protein-1 (XBP1), CCAAT/enhancer-binding protein–homologous protein (CHOP), NF-κB, and TXNIP signaling pathways. At the same time, mitochondrial ROS overproduction additionally contributes to NLRP3 inflammasome activation.

Upon activation, NLRP3 inflammasome enhances cellular secretion of IL-1β, IL-18, and high mobility group box 1 (HMGB1). These molecules bind to specific receptors on the endothelial cell surface, namely IL-1 receptor, IL-18 receptor, and TLR2/4, activating myeloid differentiation factor 88 (MyD88), IL-1 receptor-associated kinase 1/4 (IRAK1/4) and TNF receptor-associated factor (TRAF) [24]. Ultimately, the subsequent NF-κB activation promotes the formation of secondary inflammatory molecules, such as TNF-α and IL-6, which triggers the hepatic production of C reactive protein, fibrinogen, and plasminogen activator inhibitor 1 (PAI-1). At the same time, NF-κB activation assists leukocyte mobilization via upregulation of adhesion molecules (intercellular adhesion molecule 1 (ICAM-1), vascular cell adhesion molecule 1 (VCAM-1), P-selection, and E-selectin) and chemokines (monocyte chemoattractant protein 1 (MCP-1)). Moreover, HMGB1 can also bind to the receptor for advanced glycation end products (RAGE), triggering p38 MAPK activation, phosphorylation of the actin-binding protein Hsp27 and caldesmon, leading to actin stress fibers formation, cytoskeletal remodeling, and endothelial contraction. Therefore, apart from the increased leukocyte transmigration to the vessel wall, there is an enhanced endothelial cell permeability. Lastly, this pro-inflammatory state could induce endothelial cell senescence and secretion of senescence-associated secretory phenotype (SASP), limiting endothelial cell proliferation and promoting vascular sclerosis.

The ultimate result of those deleterious processes associated with NLRP3 inflammasome activation is atherosclerosis progression. Atherosclerotic plaques from carotid artery specimens were found with increased expression of NLRP3 signaling pathway molecules (NLRP3, ASC, caspase-1, IL-1β, and IL-18) compared to non-atherosclerotic arteries [25,26]. In the coronary tree, upregulation of ASC, caspase-1, and IL-18 was noted in segments with advanced atherosclerosis, together with prevalent NLRP3 inflammasome-positive foam cells around the necrotic core [27]. Clinically, NLRP3 inflammasome expression has been found to increase in patients with an acute coronary syndrome, followed by patients with stable angina, compared to those without coronary artery disease [28,29,30], indicating an association with plaque vulnerability and clinical events.

Moving downstream in the NLRP3 inflammasome pathway, IL-1 is a major mediator of inflammation in various pathologic states, such as autoinflammatory diseases (familial Mediterranean fever), acute infections, and chronic inflammatory environments, such as cancer and atherosclerosis [31]. In the latter, it promotes its initiation by activating the vascular endothelium, with subsequent upregulation of adhesion molecules, mobilization and transmigration of leukocytes [32]. IL-1 is also produced by cells found in the atheroma following inflammatory stimuli, and, thus, its role in atherogenesis progression was assumed and later demonstrated in animal studies [33,34]. This was accomplished via pharmaceutically or genetically augmenting or limiting IL-1 function. In pig models, IL-1β caused the thickening of the intima of coronary arteries and vasospasm in vivo [35]. Furthermore, IL-1 causes the autocrine production of platelet-derived growth factor (PDGF), which, in turn, stimulates human VSMC proliferation. Besides VSMCs, IL-1 modulates cardiac myocyte functionality and may hinder cardiac contractility and remodeling post-myocardial infarction. IL-1 can also act on endothelial cells, leukocytes, and VSMC to potentiate the production of inflammatory mediators (IL-6, TNF-α, CCL2), potentiating its anti-inflammatory effect. Ultimately, molecules that are responsible for plaque remodeling (MMP-3), erosion (MMP-2, -9), and rupture (MMP-1, -8, -13) are upregulated [36].

Early reports from Frostegård et al. indicated the presence of IL-1α and IL-1β in advanced human atherosclerotic plaques [37]. However, in the initial stages of atherosclerosis, IL-1α may be absent [38]. Interestingly, an experimental study using (99m)Tc-TNFR2-Fc-IL-1RA single photon emission tomography in ApoE^−/−^ and ApoE^+/+^ mice fed with either atherogenic or normal diet demonstrated greater uptake of the radiotracer in the ApoE^−/−^ mice and those on an atherogenic diet [39]. These findings correlated with the real-time polymerase chain reaction that found an increased expression of IL-1β in atherosclerotic mice aortas [39]. Type 2 diabetes mellitus may also play an important role in the pro-inflammatory milieu of atherosclerosis, as shown by the increased IL-1β mRNA in atherosclerotic plaques of patients with type 2 diabetes compared to non-diabetic plaques [40]. In another study, IL-1α was independently associated with a high vulnerable plaque burden after a multivariable analysis in 301 patients undergoing coronary computed tomography angiography (CCTA) [41]. Another study reported that NLRP3 inflammasome drives the production of equal amounts of IL-1α and IL-1β in the atherosclerotic plaque, and IL-1β concentration is higher in individuals with complex plaques [42]. Ragino et al. found markedly increased IL-1β expression in coronary arteries with TCFA or erosive changes compared to type 3 plaques (necrosis, degeneration, spotty calcification) [43]. Previously, it has also been suggested that overexpression of IL-1β in the atherosclerotic plaque was associated with extensive atherosclerosis [44]. A recent study shed more light on the differential effects of IL-1 isoforms in atherosclerosis [45]. IL-1α inhibition resulted in diminished outward arterial remodeling during early atherogenesis. At the same time, IL-1β inhibition had no effect on outward remodeling at all stages of atherogenesis, while also inducing an anti-inflammatory monocyte response and reduced atheroma size. IL-1β could also mediate its atherogenic action through NETosis and subsequent tissue factor overexpression [46]. To summarize, IL-1 may propagate atherosclerosis by inducing endothelial dysfunction, and assist its progression and the formation of unstable atherosclerotic plaques by IL-1β’s actions (potentiation of inflammation, enhancement of VSMC proliferation, and extracellular matrix degradation).

The other cytokines of the IL-1 family have also been investigated, albeit to a lesser degree. Data are limited regarding IL-18, another downstream product of NLRP3 inflammasome activation. IL-18 may promote endothelial cell dysfunction and initiate the deleterious atherosclerotic cascade, as well as promote plaque progression and destabilization by increasing MMPs and inducing IFN-γ expression from macrophages and VSMCs [47]. Moreover, IL-18 can potentiate the synthesis of IL-1α, IL-1β, IL-6, TNF-α, INF-γ, and CCL2 [48,49,50]. As shown by Mallat et al., unstable carotid plaques had a higher expression of macrophage IL-18 mRNA, further suggesting its possible implication in plaque instability [51]. The same was noted in coronary atherectomy specimens, with greater IL-18 immunopositivity in culprit plaques and plaques from patients with unstable angina, also correlating with IFN-γ expression [52]. A recent experimental study has suggested a link between blood levels of IL-18 and carotid artery plaque vulnerability. The highest circulating IL-18 levels were documented in models with unstable plaque, followed by stable plaque and non-plaque groups [53]. Ragino et al. reported a higher IL-18 tissue expression in coronary arteries with TCFAs or plaque erosions compared to type 3 plaques [43]. A prior clinical study found that circulating IL-18 has been associated with plaque rupture and TCFAs detected by optical coherence tomography (OCT) in 46 patients with acute coronary syndromes or stable angina pectoris [54]. Furthermore, IL-18 levels have been associated with incident coronary artery disease [55]. Despite the encouraging evidence concerning the importance of IL-18 in atherosclerotic plaque initiation (through endothelial dysfunction) and vulnerability (through extracellular matrix remodeling), we need additional information from preclinical and clinical studies to better understand these relationships.

As far as the role of IL-1Ra in unstable plaques is concerned, an experimental study using IL-1Ra^+/−^ mice showed increased atherosclerotic lesion formation compared to IL-1Ra^+/+^ mice [56]. In mice models, IL-1Ra deficiency augmented arterial inflammation and also led to aneurysm formation [57,58]. Moreover, human IL-1Ra infusion in an experimental porcine model of arterial injury displayed a significant and sustained reduction in neointima formation [59]. In human studies, the mRNA expression and the levels of IL-1Ra in the coronary ostium of culprit vessels have been found elevated [60,61], while a polymorphism in IL-1Ra that is associated with increased expression could diminish mean coronary plaque area [60].

IL-33, another member of the IL-1 cytokine family, acts as a ‘alarmin’ or stress-response cytokine that initiates and modulates an immune reaction, especially in areas that it is highly expressed, such as endothelial or epithelial cells. Its expression can be influenced by other cytokines, such as TNF-α, IFN-γ, and IL-1β [62]. When IL-33 is generated, it functions in an autocrine/paracrine way to activate the ST2L (ST2 gene-like) membrane receptor on adjacent cells, also known as the IL33R, and the IL-1 receptor-like 1 (IL1RL1) [63]. sST2, a soluble shortened version of ST2L lacking the transmembrane and intracellular domains, is produced by endothelial and immune cells either constitutively or in response to stimulation (in some cases by IL-33). sST2 is hypothesized to operate as a decoy receptor, dampening the effects of IL-33. IL-33 assumingly possesses atheroprotective properties. Several processes might account for these activities, including a change in T cell polarization from Th1 to Th2 and an increase in Treg cells, higher levels of natural IgM anti-ox-LDL antibodies [64], prevention of macrophage foam cell production [65,66], activation of type-2 innate lymphoid cells and macrophage polarization towards a ‘M2’-like, anti-atherosclerotic phenotype [67,68]. Thus, IL-33 is implicated in atherosclerosis evolution. However, proatherogenic actions have also been described in experimental studies, such as IL-33-induced tissue factor, adhesion molecules, and NF-κB expression by endothelial cells, with subsequent IL-6 and TNF-α synthesis [69,70]. At the same time, IL-33 can induce the secretion of chemokines (CCL2, CCL5) from mast cells [71]. A previous study has suggested that IL-33 may contribute to increased endothelial barrier permeability and angiogenesis [72]. Clinically, serum IL-33 concentration was increased in individuals with unstable angina pectoris and acute myocardial infarction compared to stable angina and control groups [73]. Moreover, IL-33 emerged as an independent predictor of acute coronary syndrome incidence. IL-33 was also found in abundance in the presence of vulnerable atherosclerotic plaques, and correlated with the degree of inflammatory cell infiltration [74]. However, conflicting results have also been reported, indicating IL-33′s ambiguous role in atherosclerosis [75]. A subsequent study stressed the prognostic significance of IL-33 in patients after revascularized acute coronary syndrome, with higher levels indicating a higher disease complexity and poorer 1-year prognosis [76]. It should also be stated that although the IL-33/ST2 axis may be related to coronary atherosclerosis, the various gene single nucleotide polymorphisms may modify this association, as suggested by a recently reported systematic review and meta-analysis [77]. Other than its role in atherosclerosis, IL-33 exerts a protective effect in most infectious settings, by assisting the clearance of the causal microorganism [78]. Moreover, it may exacerbate inflammatory conditions, such as asthma, chronic obstructive pulmonary disease, periodontitis, rheumatoid arthritis, and inflammatory bowel disease [78]. IL-33 is also a tumorigenic cytokine most likely through mast cell accumulation [78].

#### 3.1.2. IL-6

IL-6 is a pleiotropic cytokine that resides downstream in the IL-1 signaling cascade, is involved in the innate and adaptive immunity system, and modulates the acute-phase response and chronic inflammation. It is mostly produced after IL-1 or TNF-α triggers activated monocytes and macrophages, as well as other cell types, such as SMCs, endothelial cells, adipocytes, fibroblasts, and T helper 2 cells [79]. IL-6 increases the formation of acute phase reactants such fibrinogen, plasminogen activator inhibitor, which prevents fibrinolysis, and CRP by binding to membrane-bound IL-6 receptors (IL-6R) on hepatocytes in the classical signaling pathway [80]. The IL-6/IL-6R complex then binds to the two subunits of the membrane-bound gp130, forming a hexamer and promoting intracellular signaling [80]. Even though most cells do not express IL-6R on their membrane and cannot respond to IL-6 stimulation, endothelial cells represent an exception to this rule, together with lymphocytes, macrophages, and hepatocytes. IL-6 can also bind to soluble IL-6R in tissues and serum, allowing signaling in most cell types (trans-signaling) [80]. The production of soluble IL-6R is based on the cleavage of membrane-bound IL-6 by ADAM10 (a disintegrin and metalloproteinase domain-containing protein 10) and ADAM17 [81]. Recently, a third signaling method has been described, based on the interaction between dendritic cells and T cells (trans-presentation) [82]. After binding to gp130, the intracellular Janus kinase/Signal transducer and activator of transcription 3 (JAK/STAT3) signaling pathway is activated, ultimately leading to specific gene expression. As a contributor to inflammation, IL-6 is implicated in the pathogenesis of related diseases, such as rheumatoid arthritis, solid organ and hematologic malignancies [83].

IL-6 may potentiate the initiation and development of atherosclerosis by inducing platelet and coagulation cascade activation, upregulation of adhesion molecules expression, and loss of endothelial layer integrity [84,85,86,87]. It can further augment the inflammatory response by stimulating the production of TNF-α, CXCL8, and CCL2 [88]. In confirmation of its atherogenic action, a seminal experimental study detected an increase in atherosclerotic lesion size in mice treated with recombinant IL-6 compared to saline [89]. In the presence of plaque instability, the in situ IL-6 expression is also elevated, as seen in immunohistochemistry assessment of unstable carotid plaque specimens compared to stable plaques [90,91]. A reduction in IL-6 level at the plaque may lead to stabilization [92]. Ragino et al. found a higher coronary IL-6 expression in the presence of TCFAs or plaque erosions compared to type 3 plaques [43].

In clinical studies, IL-6 consistently associates with an increased risk of future non-fatal myocardial infarction and coronary artery disease, even larger than cemented cardiovascular risk factors, such as blood pressure and LDL [55]. Peripheral blood IL-6 has been considered an independent predictor of OCT-defined TCFAs, with superior diagnostic accuracy compared to high sensitivity CRP (area under the receiver operating characteristics curve (AUROC_IL-6_): 0.783 vs. AUROC_hs-CRP_: 0.715) [93]. Recently, a study suggested that IL-6 levels were elevated in 69 coronary artery disease patients with TCFAs and high-risk plaques, as defined by OCT and intravascular ultrasound [94]. Another large-scale study conducted by Ferencik et al. demonstrated that IL-6 was related to Coronary Artery Disease Reporting and Data System (CAD-RADS) categories and the degree of coronary stenosis in CCTA, being also predictive of major adverse cardiovascular events [95]. Another interesting observation was made by Bambrough et al., who identified a local biomarker signature, the IL-6 trans-myocardial gradient, prior to percutaneous coronary intervention as an important indicator of plaque burden and minimal lumen area [96]. In this direction, it has been previously reported that aspirated serum in culprit coronary arteries of patients with acute myocardial infarction had significantly higher concentrations of IL-6 compared to peripheral blood early in the course of the event [97].

Apart from the clinical data supporting the association of IL-6 with atherosclerotic diseases, there is a genetic background in this interaction. To begin with, the *IL6R* locus has been detected in genome-wide association studies of coronary artery disease [98]. Moreover, genetic alterations in IL-6 signaling increase the cleavage of membrane-bound IL-6R, thus being positively correlated with plasma IL-6R, CRP, and cardiovascular risk, according to mendelian randomization studies [99,100]. Lastly, the genetic variation in *IL6R* was associated with atherosclerotic manifestations, such as coronary artery disease, peripheral artery disease, and aortic aneurysms [101].

#### 3.1.3. IL-10

IL-10 is a model anti-inflammatory cytokine produced largely by Th2 macrophages and Treg cells. IL-10 has been implicated in numerous pathologies, including a beneficial effect in allergic states, autoimmunity, and infections [102]. At the same time, its role in neurodegenerative diseases and malignancies is controversial [103,104]. In atherosclerosis, it exerts its protective effects via inhibition of inflammation (suppression of IL-1, IL-6, and TNF-α), oxidative stress, endothelial monocyte adhesion, and lesional foam cell apoptosis [105,106,107,108]. Moreover, IL-10 can modulate lipid metabolism by promoting macrophage lipid uptake and reverse cholesterol transport [109]. Other than preclinical studies indicating a role in atherosclerosis, Treg cells and IL-10 were found in lesser concentrations in patients with a prior myocardial infarction [110]. However, tissue expression of IL-10 might differ from circulating one, as shown by Nishihira et al. in an immunohistochemical study of coronary lesions [111]. As a biomarker, IL-10 was assessed in 930 participants without cardiovascular disease in the Multi-Ethnic Study of Atherosclerosis. The investigators noted no associations with cardiovascular outcomes or coronary artery calcium during an average 10.2-year follow-up [112]. Interestingly, a study in patients with non-ST-elevation myocardial infarction found that the highest quartile of IL-10 concentration was associated with poor cardiovascular outcomes [113]. However, these results are contradictory to a previous study in a similar patient population [114]. In another recent study, higher IL-10 was associated with a lower prevalence of severe coronary artery disease in patients with metabolic syndrome [115]. In patients with human immunodeficiency virus, IL-10 was inversely correlated with total and noncalcified coronary plaque volume [116]. The variation in the results of human studies suggests that circulating IL-10 levels probably fluctuate according to the clinical context.

### 3.2. TNF-α

TNF-α acts as a ligand for two TNF-α receptors (TNFRs), TNFR1 and TNFR2. TNFRs are single transmembrane glycoproteins with extracellular TNF-binding domains that are distinguished by four tandemly repeated cysteine-rich motifs [117]. TNFRs are normally found on the cell membrane, but they can be liberated in soluble forms, capable of binding and neutralizing the action of circulating soluble TNF-α. Most cells in the body express TNFR1 on a constant basis. TNFR2 expression, on the other hand, is often stimulated by pro-inflammatory stimuli and is primarily limited to immune cells, but it can also be induced by endothelial cells or cardiomyocytes [118]. The bioavailability of the soluble and membrane-bound versions of TNF-α is crucial for TNFR1 or TNFR2 activation. Soluble TNF-α has a much higher affinity for TNFR1, whereas membrane-bound TNF-α primarily activates TNFR2 [119]. The activation of TNFR1 and TNFR2 causes a differential molecular response in the afflicted cell, leading to various effector outputs. Furthermore, because membrane-bound TNF-α is capable of conveying reverse signals, it could also be regarded as a receptor [120], in which case TNFRs, membrane-bound and soluble, can act as ligands. TNFR2 is primarily responsible for triggering membrane-bound TNF-α reverse signaling [120]. The role of TNF-α in autoimmune diseases (rheumatoid arthritis, psoriasis, inflammatory bowel disease) is well-described [121], while it has dual function in cancer development [122]. TNF-α can also induce the expression of other major inflammatory mediators, such as IL-1α, IL-1β, IL-6, and CCL5, thus enhancing the inflammatory effect [123,124].

The role of TNF-α in atherosclerosis has been documented experimentally. Initially, there was histological evidence of the presence of TNF-α in macrophages, VSMCs, and other cells in the intimal layer, as well as in pultaceous-rich plaques [125]. Moreover, carotid plaques from symptomatic patients have a higher TNF-α concentration compared to the plaques of asymptomatic individuals [126]. The atherogenic potential of TNF-α was highlighted in an experimental study including TNF-α and ApoE double-knockout mice. Compared to ApoE^−/−^ mice, the double-knockout group exhibited a smaller atherosclerotic plaque area and lesion size in the aorta, together with downregulated adhesion molecule expression. Interestingly, macrophages in the double-knockout group displayed a lower uptake of oxLDL and increased expression of the scavenger receptor class A [127]. In another study, mice lacking TNF-α on an APOE*3-Leiden background had a higher burden of early lesions, while the advanced lesions had enhanced necrosis and diminished apoptosis, compared to APOE*3-Leiden only mice [128]. The main mechanisms implicated in TNF-α-induced atherogenesis are endothelial dysfunction, oxidative stress, and VSMC secretory phenotype promotion [129]. In human umbilical vein endothelial cells, TNF-α enhanced LDL transcytosis and retention in the subendothelial space, thus aiding the atherosclerotic cascade [130]. A recent study has shown that TNF-α production by B2 cells is responsible for macrophage-induced cell death and inflammation, thus leading to atherosclerotic plaque vulnerability [131]. Additionally, it should be noted that circulating TNF-α levels reflect plaque TNF-α concentration, as documented by Edsfeldt et al. in an experimental study [132].

According to the evidence mentioned above, it could be suggested that TNF-α is an active mediator of plaque vulnerability, as shown by the study of Ragino et al. who detected similar coronary TNF-α expression in coronary arteries with different types of instability features (TCFA, erosion, neurodegeneration-necrosis-calcification) [43]. In the clinical setting, it has long been shown that increased circulating TNF-α concentrations may signify an excess risk of recurrent cardiovascular events in patients after myocardial infarction [133]. The association of circulating TNF-α with incident coronary artery disease in a population-based cohort was confirmed in the study of Kaptoge et al. [55]. The study group also proceeded to a meta-analysis of the available evidence (29 studies), demonstrating an increased risk for non-fatal myocardial infarction or cardiovascular mortality per 1 standard deviation increased TNF-α levels (adjusted hazard ratio: 1.17, 95% confidence intervals 1.09–1.25) [55]. TNF-α has been associated with IVUS-defined plaque burden and TCFAs in patients with stable angina [134]. Recently, high levels of soluble TNFR1 and TNFR2 have also been associated with major adverse cardiovascular events in stable coronary artery disease patients, despite not adding incremental risk prediction [135].

### 3.3. Interferon-γ

IFN-γ communicates via a heterodimeric cell-surface receptor composed of two distinct subunits: IFN-γ receptor (IFNGR)-1 and IFNGR2 [136]. Following IFN-γ interaction, the receptor subunits heterodimerize, activating JAK1 and JAK2, which phosphorylate their downstream substrate, STAT-1. STAT1 homodimerizes and binds to activated regions, causing transcription to occur. IFN-γ is involved in anti-tumorigenic and pro-tumorigenic effects [137], while also influencing autoimmunity [138]. IFN-γ is crucial in the initiation of atherogenesis, since it promotes endothelial cell activation and leukocyte mobilization [136]. Among the other atherogenic actions of IFN-γ are the potentiation of inflammation (upregulation of IL-1β, IL-6, and CCL5, downregulation of IL-1Ra) shift of macrophages towards the atheroprone M1 phenotype, the polarization of T cells towards a Th1 phenotype, and the inhibition of collagen synthesis, ultimately resulting in plaque destabilization [124,136,139,140]. INF-γ may also synergize with TNF-α to produce a more potent pro-inflammatory effect [141]. Experimental studies in mice deficient for IFN-γ receptor or IFN-γ have consistently detected a reduction in atherosclerotic lesion size, lesion lipid area and cellularity, paired with an increased collagen content, indicative of plaque stability [142,143]. Moreover, daily injections of IFN-γ in ApoE^−/−^ resulted in increased lesion size and T-cell accumulation [144]. Clinical data is scarce, with one study showing an increased concentration of IFN-γ in patients with unstable angina pectoris compared to stable angina pectoris and the control group [145], indicative of INF-γ’s plaque destabilizing properties.

### 3.4. Chemokines

Several chemokines have been described in the development of coronary atherosclerosis due to their potential to regulate blood cell migration, arrest on endothelium, and endothelial transmigration. To begin with, CCL5, also known as RANTES, promotes monocyte recruitment through binding to its membrane-bound receptors CCR5 and CCR1, or other soluble chemokines (CXCL4, CCL17, CXCL12) and defensins (human neutrophil peptide 1). Protein and mRNA levels of RANTES have been positively associated with vulnerable plaque morphology in an experimental rabbit model [146]. Indeed, higher RANTES levels were related to the carotid wall thickness and lipid core volume in participants of the Atherosclerosis Risk in Communities study [147]. Moreover, coronary artery disease severity was correlated with RANTES, since higher levels were noted in patients with acute myocardial infarction and unstable angina pectoris compared to stable angina pectoris [148]. RANTES also participates in the entire spectrum of liver diseases [149], as well as in malignancies [150,151]. Moving to the CCL2/CCR2, it acts to attract circulating monocytes and contributes to the egress of monocytes from the bone marrow, as well as stimulating IL-6 production [152]. Genetically predicted levels of CCL2, also known as MCP-1, relate to a higher coronary artery disease and stroke risk, as shown in a recent mendelian randomization study [153]. In addition to this finding, a meta-analysis of 7 cohort studies with 21,401 participants free of cardiovascular disease documented an association of higher circulating MCP-1 levels with long-term cardiovascular mortality [154]. The tissue concentration MCP-1 has also been found elevated in the presence of a TCFA or erosive changes compared to neurodegenerative necrosis of the coronary plaque [43]. Other than its cardiovascular implication, MCP-1 orchestrates cancer progression [155], and is involved in infections, neurogenerative diseases, liver diseases, rheumatoid arthritis, inflammatory bowel disease, and central nervous system pathologies, among others [156].

Other molecules with less well-established effects in atherosclerosis have also been reported. The membrane-bound CX_3_CL1 and CXCL16 bind to CX_3_CR1 and CXCR6, respectively, but can also be proteolytically cleaved by ADAMs, aiding leukocyte migration to sites of injury. CXCL16 can scavenge oxLDL, thus antagonizing atherosclerosis progression [157]. Beyond atherosclerosis, CXCL16 is involved in non-alcoholic fatty liver disease, inflammatory bowel disease, human immunodeficiency virus disease course, as well as tumorigenesis [158]. On the other hand, CX_3_CL1 orchestrates macrophage-VSMC cross-talk [159], platelet activation, and aggregation [160], indicating a possible role in atherosclerosis progression other than initiation. CX_3_CL1 levels may be associated with coronary artery disease severity, since elevations were observed in patients with acute myocardial infarction and unstable angina pectoris compared to stable angina pectoris [148]. Circulating CX_3_CL1 also correlated with plaque burden on intravascular ultrasound [161], as well as with plaque rupture in patients with unstable angina pectoris [162], providing confirmation of its potential role in the progression of coronary artery disease. Besides, the CX_3_CL1/CX_3_CR1 axis is involved in the pathophysiology of multiple other inflammatory, infectious, and neurological disorders, as well as several types of cancer [163]. The role of CXCR2, CXCR3, and their ligands, as well as the CXCL12/CXCR4 axis, is ambiguous and has not been clearly defined.

## 4. Cytokine-Targeted Strategies in Coronary Artery Disease

It is now known that lowering the inflammatory burden is of similar importance to lipid-lowering in terms of reducing atherosclerotic complications, and managing both the inflammatory and dyslipidemic components could lead to the greatest benefit [164]. Many of the readily available pharmacologic options possess potent anti-inflammatory and cytokine-lowering properties, including statins and sodium-glucose cotransporter 2 inhibitors [165,166,167,168,169]. Both drug categories were also proven to prevent atherosclerosis progression and promote plaque stabilization [170,171,172]. Most notably, in the landmark JUPITER trial, rosuvastatin led to a reduction in major adverse cardiovascular events in patients with low LDL-C but high CRP (>2 mg/L) [164], with the greatest benefit being observed in subjects achieving LDL-C <70 mg/dL and CRP <1 mg/L [173]. Methotrexate (MTX), a broad-based immunomodulatory agent, has also received attention since it could mediate vascular inflammation, endothelial protection, and lipoprotein transportation preclinically, thus inducing plaque regression [174,175,176,177]. However, in the seminal CIRT trial, there was no effect of low-dose weekly MTX on major adverse cardiovascular events compared to the placebo [178]. It should be stated that the lack of a pro-inflammatory patient profile based on the levels of inflammatory biomarkers could have accounted for those neutral results.

**Figure 2:**
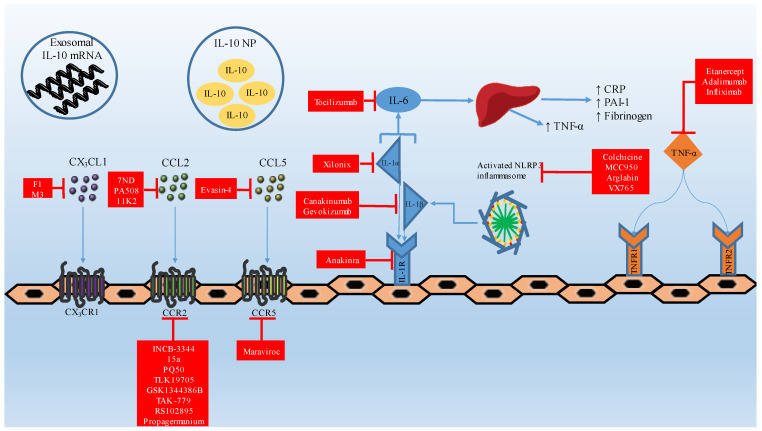
Cytokine-based therapeutics in coronary atherosclerotic disease. IL: interleukin; NP: nanoparticle; IL-1R: interleukin-1 receptor; TNF-α: tumor necrosis factor-α; CRP: C reactive protein; PAI-1: plasminogen activator inhibitor-1; TNFR: tumor necrosis factor receptor.

Since the inception of the notion that inflammatory components contribute to the atherosclerotic process, several cytokines and their pathways have been specifically targeted to evaluate their effect in preventing atherosclerosis. Herein we discuss the available therapeutic options based on the results of clinical trials, while also providing an overview of the current experimental landscape in immunomodulatory therapy of coronary artery disease (Figure 2).

### 4.1. Colchicine

Colchicine, a low-cost anti-inflammatory agent that is widely used in pericarditis, gout, and familial Mediterranean fever, and has been shown to suppress the production of IL-1β, IL-6, and IL-18 [179]. Colchicine has a modulatory effect on the NLRP3 inflammasome. Colchicine treatment may result in lower extracellular vesicle NLRP3 protein levels in vivo [180]. In patients with an acute coronary syndrome, colchicine limited IL-1β secretion and intracellular content, while also diminishing pro-caspase-1 mRNA and secreted caspase-1 protein levels [181]. NLRP3 inflammasome formation, caspase-1 activation, and IL-1β production were also previously found to be suppressed by colchicine experimentally [182].

In patients with an ST-elevation myocardial infarction, treatment with colchicine for 5 days resulted in lower absolute and relative infarct size [183]. These observations were followed by large-scale clinical trials. In the COLCOT randomized, double-blind trial, colchicine’s effectiveness was examined for the secondary prevention of cardiovascular events in 4745 patients with a history of acute myocardial infarction within 30 days and a left ventricular ejection fraction greater than 35% [184]. Almost all patients were under both aspirin with another antiplatelet agent and a statin. The results showed that colchicine at a dose of 0.5 mg daily led to a significantly lower risk of ischemic cardiovascular events when compared to the placebo (hazard ratio 0.77, 95% confidence interval: 0.46–1.52), but no change in overall survival (hazard ratio 0.98, 95% confidence interval 0.64–1.49). Moreover, earlier initiation (within 3 days) was associated with incremental clinical benefits [185]. In the other seminal trial, LoDoCo-2, 5522 patients with chronic coronary artery disease were randomized to placebo or colchicine 0.5 mg once daily [186]. The primary endpoint of cardiovascular death, spontaneous myocardial infarction, ischemic stroke, or ischemia-driven coronary revascularization occurred less frequently in the colchicine arm (hazard ratio 0.69, 95% confidence interval 0.57–0.83) during a median follow-up of 28.6 months. This benefit was irrespective of prior acute coronary syndrome and continued to accumulate even after 5 years of follow-up [187,188]. Finally, a meta-analysis has demonstrated the cardiovascular benefits of colchicine in acute and chronic coronary artery disease [189]. Regarding its potential side effects, colchicine may increase the risk of diarrhea, without significantly enhancing the risk of infections, liver, and hematological diseases [190].

### 4.2. IL-1 Inhibition

The potential of IL-1β manipulation in atherosclerosis has been known for years, since ApoE^−/−^/IL-1β^−/−^ mice exhibited significant decreases in aortic sinus atherosclerotic lesions and adhesion molecules expression compared to ApoE^−/−^ only mice [191]. However, validation in the form of a clinical trial was only provided recently. IL-1β may be selectively inhibited by canakinumab, a fully human monoclonal antibody which is actively used in rheumatologic disorders, such as systemic juvenile idiopathic arthritis and Still’s disease. Canakinumab binds to IL-1β with high affinity. The antigenic epitope includes Glu 64, which is essential for the recognition of human IL-1β by the antibody. Canakinumab’s main advantage is that it reduces plasma levels of IL-6 and hsCRP, leaving LDL unaffected. Thus, it was hypothesized that its potential effect on cardiovascular events would be independent of lipid-level lowering, which is the gold-standard strategy for cardiovascular event prevention. Another member of this class, gevokizumab, reduced carotid intima-media thickness and neointima formation in an experimental model of rat carotid denudation, paired with improved endothelial regeneration [192].

The CANTOS study was a randomized, double-blind clinical trial exploring the effect of canakinumab as a proof-of-concept anti-inflammatory therapy in the recurrence of cardiovascular events, compared to the placebo [193]. The trial population consisted of 10,061 stable patients with a history of myocardial infarction and elevated high-sensitivity CRP (≥2 mg/L). The patients had a baseline LDL-cholesterol of 82 mg/dL and a median high sensitivity CRP of 4.1 mg/L. The patients in the canakinumab group received the drug at a dosage of 50, 150, or 300 mg once every 3 months. The results were encouraging, since non-fatal MI, non-fatal stroke or cardiovascular death were reduced by 15% over a median follow-up of 3.7 years. Furthermore, inflammatory markers, such as hsCRP and IL-6, demonstrated a dose-dependent reduction. Participants who achieved IL-6 levels below the study median (1.65 ng/L) after the first dose experienced the greatest benefit in several pre-specified endpoints, while those with consistently higher values accrued no benefit [194]. However, canakinumab’s use may be accompanied by significant side effects (increased risk of fatal infections, leukopenia, thrombocytopenia), while its high cost does not allow for widespread clinical application in the setting of secondary prevention. It should also be stressed that the levels of IL-18 remained unchanged after canakinumab treatment. Since baseline and on-treatment levels of IL-18 were associated with future cardiovascular events, the subset of patients with persistently elevated IL-18 remains at risk despite canakinumab treatment [195].

The role of IL-1α in atherosclerosis is, as stated previously, has been less straightforward, thus its inhibition is not broadly attempted. In the only phase II study in an atherosclerotic setting to date, Xilonix, a true human monoclonal antibody that specifically targets IL-1α, was administered parenterally in subjects after percutaneous lower limb revascularization [196]. This trial, although proving Xilonix’s safety, was neutral in terms of efficacy. Specifically, the rates of major clinical events and restenosis were similar between the two study arms, with a significant difference being noted only in the intravenous dosing period. Thus, the role of IL-1α inhibition in atherosclerosis is uncertain.

### 4.3. IL-6 Inhibition

Since the IL-6 receptor has been considered a causal factor in coronary artery disease development and progression [99], its inhibition has been attempted. Preclinically, tocilizumab, a humanized monoclonal antibody against the IL-6 receptor, abrogated hyperglycemia-induced VSMC migration, adhesion molecule, and MMPs expression [197]. Moreover, it may ameliorate endothelial glycocalyx dysfunction and oxidative stress, which are important drivers of atherogenesis [198]. It should be noted that tocilizumab may augment total and LDL cholesterol [199]. However, it can limit LDL’s pro-atherogenic potential by increasing HDL’s cholesterol efflux capacity [200]. Another study has shown that tocilizumab could decrease lipoprotein (a) and oxLDL concentrations, without, however, affecting cholesterol efflux capacity [201]. Additionally, tocilizumab may attenuate platelet aggregation, as recently shown [202]. In patients with non-ST-elevation myocardial infarction, tocilizumab was associated with suppressed C5aR1 and the C5aR2 mRNA levels, receptors that are associated with pro-inflammatory signaling [203].

In the landmark ASSAIL-MI trial of tocilizumab in 199 patients with ST-elevation myocardial infarction, patients in the study group had a greater myocardial salvage index compared to the control group [204], indicating lesser myocardial/reperfusion injury. This outcome could stem from the reduction of neutrophil count and impaired neutrophil function with tocilizumab [205]. The action of tocilizumab’s is not straightforward, as a placebo-controlled trial in patients with non-ST-elevation myocardial infarction suggested that it may potentiate NETosis [206]. According to the ASSAIL-MI trial, no significant infectious side effects were attributed to tocilizumab use when compared to the placebo, even though the available evidence concerns only short-term follow-up [204]. However, in individuals with rheumatological conditions, the use of tocilizumab was associated with a significantly higher risk of serious infections [207]. Future trials and prospective cohort studies should also assess IL-6 receptor antagonism in primary and secondary prevention of major adverse cardiovascular events of high-risk patients.

### 4.4. TNF-α Inhibition

TNF-α inhibition could represent an appealing anti-inflammatory approach in the setting of atherosclerosis. However, the effect of TNF-α inhibitors, such as etanercept, adalimumab, and infliximab, in atherosclerosis has been evaluated mostly preclinically or in studies of patients with rheumatological diseases. TNF-α inhibitors have shown anti-inflammatory and endothelial-protective properties. Incubation of human coronary artery endothelial cells with inflammatory stimulants (IL-1β, TNF-α, serum amyloid A) promoted an increase in inflammatory cytokines which was abrogated by infliximab treatment [208]. Etanercept prevented TNF-induced endothelial cell apoptosis by enhancing the process of autophagy [209]. Moreover, infliximab reversed TNF-α induced endothelial dysfunction as well as the increase in oxLDL, LOX-1, and Arg2 [210]. We have also shown that adalimumab could improve endothelial function and inflammation in patients with psoriasis [211]. Moreover, the vasculoprotective effects of TNF-α inhibitors include the amelioration of arterial stiffness [212].

The effect of TNF-α inhibition in the prevention of major adverse cardiovascular events has been controversial. Earlier studies in patients with psoriasis or rheumatoid arthritis pointed to a lower incidence of hard cardiovascular endpoints with TNF-α inhibitors [213,214]. However, in a prospective cohort study of patients with psoriasis, the rate of major adverse cardiovascular events did not differ between TNF-α inhibitors, methotrexate, or ustekinumab [215]. The increased risk of serious infections with TNF-α inhibitors should not be neglected [216]. Based on the available information, it is certain that more evidence is needed in the assessment of TNF-α inhibitors in coronary artery disease.

### 4.5. IL-1Ra

The recombinant, non-glycosylated form of the human IL-1Ra, anakinra, prevents IL-1 binding to the IL-1R1. Due to its lack of the necessary binding domain for downstream signaling, it is efficacious in reducing inflammation. In cardiovascular disease, this agent is mainly employed as an additional tool in the management of pericardial diseases [217]. Limited data are available regarding its potential role as an anti-atherosclerotic treatment. In diabetic rats, anakinra partially ameliorated endothelial dysfunction and oxidative stress parameters, which are important steps in the atherosclerotic cascade [218]. Anti-atherosclerotic actions were recently reported in ApoE^−/−^ mice treated with anakinra, such as reduced aortic arch atherosclerotic lesions and lower inflammatory gene expression [219]. In the clinical setting, anakinra could exert anti-inflammatory actions in patients with ST-elevation myocardial infarction [220]. However, it is unclear whether recombinant IL-1Ra could provide incremental benefits in the prevention of major adverse cardiovascular events [221]. It should also be noted that, as with other IL-based therapeutics, the risk of serious infections may be higher with anakinra [207].

### 4.6. Future Cytokine-Based Therapeutics

Several agents are currently under investigation in preclinical studies of atherosclerosis, as summarized in Table 2. As stated above, the upstream target in IL-1β pathway is the NLRP3 inflammasome. Therefore, its pharmacological targeting could represent a valid option in the management of atherosclerotic diseases. Experimental molecules have been developed with inhibiting properties, including MCC950, arglabin, and VX765. Beginning with MCC950, it was able to alleviate myocardial ischemia/reperfusion injury in a porcine model of myocardial infarction by limiting neutrophil influx into the myocardium and IL-1β levels [222]. Subsequently, in an ApoE^−/−^ mouse model of carotid atherosclerosis, MCC950 induced a reduction in maximal stenosis, average plaque size, and plaque volume, along with diminished macrophage accumulation and adhesion molecule mRNA expression [223]. Moreover, infusion of platelet-derived extracellular vesicles loaded with MCC950 in ApoE^−/−^ mice abrogated atherosclerotic plaque formation, through the reduction in inflammation, macrophage proliferation, and T cell localization in the plaque [224]. An interesting study was performed by Sharma et al. in ApoE^−/−^ mice with streptozocin-induced diabetes [225]. In this deleterious setting, NLRP3 inflammasome activation was more evident, but was halted by the MCC950 administration. Moreover, this agent attenuated numerous atherogenic processes, including oxidative stress, inflammation, macrophage accumulation, necrotic core formation, and fibrous cap thinning [225]. Improvement in macrophage pyroptosis and inflammation with MCC950 was also proven in the study of Zeng et al. [226]. In the most recent study of MCC950, it prevented leukocyte migration through lower chemokine and adhesion molecule expression from endothelial cells [227]. Interestingly, MCC950 attenuated leukocyte production by the bone marrow. Arglabin has been investigated scarcely in atherosclerotic studies, promoting anti-inflammatory (anti-inflammatory macrophage phenotype switching) and hypolipidemic (reduction in total cholesterol and triglycerides) effects [228]. These were accompanied by the prevention of atherosclerotic lesion expansion. Finally, VX765, a caspase-1 inhibitor prodrug activated by intracellular esterases, attenuated VSMC pyroptosis and atherosclerosis progression in ApoE^−/−^ mice on a Western diet [229].

Moving to IL-10 targeting, several studies have assessed the therapeutic value of its overexpression towards atherosclerosis. Initial studies utilized viral vector-mediated interleukin-10 gene transfer in ApoE^−/−^ mice. A decrease in inflammatory mediators was observed, together with a reduction in macrophage infiltration, leading to a lower lesion size [105,230]. A recent study proceeded to the delivery of nanoparticles incorporating IL-10 (Col-IV IL-10) in a mouse model of advanced atherosclerosis (LDLr^−/−^ on a high-fat diet), with the investigators noting enhanced fibrous cap thickness and attenuated necrotic core formation [231]. In comparison to free IL-10 administration, nanoparticle delivery of IL-10 in the atherosclerotic plaque produced a greater anti-inflammatory effect and significant atherosclerotic lesion regression [232]. Furthermore, exosome-mediated delivery of IL-10 mRNA in atherosclerotic plaque promoted its efficient translation by the various cells and M1 macrophages. IL-10 protein was then retained in the plaque and acted accordingly to attenuate atherosclerosis progression [233].

Anti-IFN-γ therapies have been attempted preclinically, since their clinical use is currently limited by the feared side effects, namely infections. In ApoE^−/−^ mice on a high-fat diet, soluble mutant IFN-γ receptor injection every 2 weeks resulted in decreased lesion size, lipid and macrophage area, as well as a higher degree of fibrosis and VSMC content, both at the early and later stages of the atherosclerotic cascade [234,235].

In the field of anti-chemokine therapeutics, the blockade of RANTES receptors attenuated atherosclerosis, as shown by impaired leukocyte migration and plaque stabilization [242]. Maraviroc, a CCR5 antagonist, ameliorated endothelial function, platelet-leukocyte aggregate formation, and carotid intima-media thickness in persons with human immunodeficiency virus [236]. Preclinically, this agent also attenuated VSMC proliferation and phenotypic transformation into a synthetic cell type [237], apart from its anticipated role in limiting monocyte recruitment into the arterial wall [243]. Evasin-4, a RANTES inhibitor, reduced myocardial infarct size, leukocyte recruitment, and oxidative stress while also improving survival in wild-type mice subjected to left coronary artery ligation [238]. Modulation of the CCL2/CCR2 axis may constitute a fascinating approach against atherogenesis. A recent meta-analysis of preclinical studies has been published, summarizing the scientific evidence in this pathway [239]. CCL2/CCR2 inhibition attenuated atherosclerotic lesions in the aorta, carotid, and femoral arteries. Moreover, it increased collagen deposition and smooth muscle cell content, together with a decrease in macrophage accumulation. The effect of this approach on lesion size and macrophage accumulation was consistent regardless of lesion stage at onset, diet plan, pharmacologic target (CCL2 or CCR2), and mouse model. Last but not least, inhibition of the CX_3_CL1/CX_3_CR1 axis may represent another valid approach. F1, a modified CX_3_CR1 ligand, ameliorated atherosclerotic lesion development in ApoE^−/−^ and LDLr^−/−^ mice through a reduction in macrophage accumulation and inflammatory infiltration [240]. The effect was retained even in an advanced atherosclerosis setting. M3, a compound that involves CX_3_CL1-inactivating properties, had inconsistent effects in atherosclerosis since it prevented lesion progression in ApoE^−/−^ on a normal chow diet while it had no effect on disease progression in the presence of a high-fat diet [241].

## 5. Conclusions

Inflammation represents a core pathophysiologic process in the evolution of atherosclerosis. In particular, the impact of cytokines in the unstable atherosclerotic plaque has been demonstrated in a variety of animal studies and, more recently, in human clinical trials. The CANTOS study provided sound evidence that targeting the IL-1 pathway for secondary prevention is indeed effective in reducing the instability of the atherosclerotic plaque. The COLCOT and LoDoCo 2 trials of colchicine in coronary artery disease further enhanced these observations.

The current challenge is the design and production of accessible drug formulations that may selectively target other cytokine pathways that are associated with residual cardiovascular risk. The effect of inhibition of the inflammasome, with a combined effect on IL-1β and IL-18, may also be examined in future animal and clinical studies. Anti-chemokine approaches are also attractive and warrant further investigation preclinically. However, such anti-inflammatory approaches in atherosclerosis should take into serious account patient safety along with efficacy measures. In this regard, a method of direct delivery into the atherosclerotic plaque through the use of nanoparticles deserves further preclinical validation.

## Figures and Tables

**Table 1:** Role and mechanism of action of various cytokines in atherosclerosis.

Cytokine	Role in Atherosclerosis	Mechanism
IL-1β IL-18	Promote	Endothelial dysfunction Monocyte migration and maturation VSMC proliferation IL-6 signaling-potentiation of inflammation ↑ MMP production
IL-1-Ra	Suppress	Prevent pro-atherosclerotic IL-1β effects
IL-33	Suppress	Change in T cell polarization from Th1 to Th2 ↑ Treg cells ↑ natural IgM anti-oxLDL antibodies ↓ macrophage foam cell production Activation of type-2 innate lymphoid cells Macrophage polarization toward a ‘M2’ phenotype
IL-6	Promote	Induction of platelet and coagulation cascade Upregulation of adhesion molecules expression Loss of endothelial layer integrity
IL-10	Suppress	↓ inflammation, oxidative stress ↓ endothelial monocyte adhesion ↓ lesional foam cell apoptosis ↑ macrophage lipid uptake and reverse cholesterol transport
TNF-α	Promote	Endothelial dysfunction Oxidative stress Potentiation of inflammation ↑ oxLDL uptake and ↓ expression of the scavenger receptor class A ↑ LDL transcytosis and retention in the subendothelial space ↑ macrophage-induced cell death and inflammation Promotion of a secretory VSMC phenotype
IFN-γ	Promote	Endothelial cell activation Leukocyte mobilization Shift of macrophages towards the atheroprone M1 phenotype Polarization of T cells towards a Th1 phenotype ↓ collagen synthesis
RANTES	Promote	Monocyte recruitment
CCL2/CCR2	Promote	Monocyte recruitment
CX_3_CL1	Promote	Monocyte recruitment Macrophage-VSMC cross-talk Platelet activation and aggregation

IL: interleukin; VSMC: vascular smooth muscle cell; MMP: matrix metalloproteinase; Ra: receptor antagonist; oxLDL: oxidized low-density lipoprotein; TNF: tumor necrosis factor; IFN: interferon; ↑: increase, ↓: decrease.

**Table 2:** Preclinical evidence of investigational cytokine-based therapies in atherosclerosis.

Agent	Target	Effect
MCC950 [222,223,224,225]	NLRP3 inflammasome	↓ inflammatory mediators ↓ neutrophil influx/migration ↓ bone marrow leukocyte production ↓ oxidative stress ↓ adhesion molecule expression ↓ macrophage infiltration and pyroptosis ↓ T cell plaque localization ↓ lesion size ↓ fibrous cap thinning
Arglabin [228]	NLRP3 inflammasome	Anti-inflammatory macrophage phenotype switching ↓ total cholesterol and triglycerides ↓ lesion size
VX765 [229]	NLRP3 inflammasome	↓ VSMC pyroptosis ↓ plaque progression
IL-10 gene transfer [105,230]	IL-10	↓ inflammatory mediators ↓ macrophage infiltration ↓ lesion size
IL-10 nanoparticles [231,232]	IL-10	↑ fibrous cap thickness ↓ necrotic core formation ↓ inflammatory mediators ↓ lesion size
Exosomal IL-10 mRNA [233]	IL-10	Prevention of atherosclerosis progression
Mutant IFN-γR [234,235]	IFN-γ	↓ lesion size ↓ lipid and macrophage area ↑ fibrosis ↑ VSMC content
Maraviroc [236,237]	CCR5	↓ monocyte recruitment ↑ endothelial function ↓ platelet-leukocyte aggregate formation ↓ VSMC proliferation ↓ VSMC phenotypic transformation into a synthetic cell type
Evasin-4 [238]	RANTES	↓ myocardial infarct size ↓ leukocyte recruitment ↓ oxidative stress
CCL2/CCR2 inhibitors [239]	CCL2/CCR2	↓ aorta, carotid, and femoral atherosclerotic lesions ↓ macrophage accumulation ↑ collagen deposition a ↑ VSMC content
F1 [240]	CX_3_CL1/CX_3_CR1	↓ atherosclerotic lesion development ↓ macrophage accumulation ↓ inflammatory infiltration
M3 [241]	CX_3_CL1/CX_3_CR1	Contradicting effect on lesion progression

VSMC: vascular smooth muscle cell; IL: interleukin; IFN-γR: interferon-γ receptor.

## Data Availability

Not applicable.
